# Higher-Dose Primaquine to Prevent Relapse of Plasmodium vivax Malaria

**DOI:** 10.1056/NEJMc2205922

**Published:** 2022-07-21

**Authors:** James A. Watson, Nicholas J. White

**Affiliations:** Mahidol Oxford Tropical Research Unit, Bangkok, Thailand

The unusual sequential “radical cure” treatment regimen for *P*. *vivax* malaria reported by Chamma-Siqueira et al. from western Brazil complicates interpretation of the trial result. Usually chloroquine and primaquine are given together to treat *P*. *vivax* malaria. Both drugs have activities at the asexual stage of parasite development, but only primaquine kills the dormant liver-stage parasites (hypnozoites) that cause relapse. Although long-latency *P*. *vivax* malaria occurs in the Americas, most relapses emerge from the liver approximately 14 days after presentation of the initial illness. The slowly eliminated chloroquine suppresses the relapsing blood-stage infection for several weeks. Chamma-Siqueira et al. delayed administration of primaquine for a median of 17 days. Because each study group received the same dose of chloroquine, the authors were therefore comparing the asexual-stage antimalarial effects of primaquine against early relapse and the hypnozoitocidal effect against later relapses. Primaquine has relatively weak activity against asexual-stage parasites, so, unsurprisingly, the 2-week course was superior to the 1-week course. Clinical trials comparing radical curative activity in *P*. *vivax* and *P*. *ovale* malaria should assess concurrent — not sequential — treatment (i.e., hypnozoitocidal efficacy and not activities at the asexual stage of parasite development should be assessed).

